# Retrospective analysis of the impact of increasing access to long acting reversible contraceptives in a commercially insured population

**DOI:** 10.1186/s12978-016-0211-3

**Published:** 2016-08-22

**Authors:** Amy Law, Dominic Pilon, Richard Lynen, François Laliberté, Laurence Gozalo, Patrick Lefebvre, Mei Sheng Duh

**Affiliations:** 1 Bayer HealthCare Pharmaceuticals Inc, 100 Bayer Blvd, Whippany, NJ 07981 USA; 2 Groupe d’analyse, Ltée, 1000 De La Gauchetière West, Bureau 1200, Montreal, QC H3B 4W5 Canada; 3 Analysis Group, Inc., 10th Floor, 111 Huntington Ave, Boston, MA 02199 USA

**Keywords:** LARC, SARC, Contraception

## Abstract

**Background:**

Unintended pregnancies have been shown to be associated with high costs for the healthcare system, among other adverse impacts, but could still account for up to 51 % of pregnancies in the US. Improvements in contraception among women are needed. Long acting reversible contraceptives (LARCs), which have proved their safety and efficacy, have been found to significantly decrease the risk of unintended pregnancy. Yet they are still marginally employed. This study aims at investigating the evolution of LARC use over 15 years and at assessing the impact of the introduction of newer LARCs on LARC use relative to all contraceptive use.

**Methods:**

This retrospective study identified women with LARC or short acting reversible contraceptive (SARC) claims from a US insurance claims database (01/1999-03/2014). Yearly proportions of LARC users relative to all contraceptive users were reported. Generalized estimating equation models were used to assess the impact of user characteristics, such as age group (15–17, 18–24, 25–34, and 35–44), and of time periods related to the introduction of new LARCs (01/2001: Mirena, 07/2006: Implanon, 01/2013: Skyla) on LARC use.

**Results:**

A total of 1,040,978 women were selected. LARC use increased yearly from 0.6 % (1999) to 16.6 % (2013) among contraceptive users. Time periods associated with the introduction of a newer LARC were significant predictors of LARC use; women in 2006-2012 and 2013-2014 were respectively 3.7-fold (95 % CI:3.57–3.74) and 6.6-fold (95 % CI:6.43–6.80) more likely to use LARCs over SARCs relative to women in 2001-2006. The increase in LARC use was especially pronounced in young women. Compared to women aged 18–24 in 2001-2006, women aged 18–24 in 2006-2012 and 2013-2014 were respectively 6.4-fold (95 % CI:5.91–6.86) and 14.7-fold (95 % CI:13.59–15.89) more likely to use LARCs over SARCs.

**Conclusions:**

This broadly representative commercial claim-based study showed that the proportion of privately insured women of childbearing age using LARCs increased over time and that the introduction of newer LARCs corresponded with significant increases in overall LARC use. Future research is needed to assess LARC use in uninsured or publicly-insured populations.

## Background

About half of the pregnancies in the United States (US) are currently unintended [1, 2]. Unintended pregnancies have been shown to have adverse social, economic, and health outcomes for the mother, and for the child in cases where the pregnancy turned into live birth [3]. Furthermore, their burden on the healthcare system could be as high as $4.5 billion [4]. Decreasing the rate of unintended pregnancies has therefore become a national public health goal [5]. In the US, according to a 2009 study, it was estimated that 43 % of unintended pregnancies were due to inconsistent contraceptive use [6]. Hence long acting reversible contraception (LARC) methods, which solve issues related to adherence and incorrect use, could significantly help in reducing unintended pregnancies and their associated burdens [7, 8].

Despite the above, LARC methods are still largely under-utilized among women [9–12]. Notably, in an analysis of national surveys, Darroch et al. [12] reported that the proportion of women using LARC methods increased between 2003 and 2012 in developing countries (from 6 to 9 %) but that it remained stable at 4 % in higher income countries. Furthermore, recent studies of the US National Survey of Family Growth (NSFG) have reported an increase in the use of LARC among women 15–44 years old from 2002 to the 2011–2013 time period [9, 11].

The main reasons identified for under-utilization of LARC methods have been misconceptions about LARC (such as safety concerns and non-eligibility of nulliparous women) at the user and provider levels, over-estimation of the efficiency of other contraceptive methods and the ability of users to optimally use them, and perceived higher costs [13]. With regards to higher costs, insurance providers play an important role in determining the consumer costs attributable to LARC use. It has been demonstrated that women with low out-of-pocket costs had a higher likelihood of choosing LARC compared to women with high out-of-pocket costs [14], and that providing complete insurance for LARC would increase its use [13].

Most of these barriers can be addressed through improved education of women and providers on LARC and contraception in general [13]. By raising awareness, providing opportunities to discuss the benefits and suitability of LARC methods, and fulfilling women’s unmet contraceptive needs by broadening the range of devices and their duration, it can be assumed that the arrival on the market of new LARC methods could contribute to this effort.

The purpose of this study was to describe the evolution of LARC use over the period 1999–2014 and to assess through the same time period the impact of introducing new LARC methods on LARC use relative to all contraceptive users in a large population of privately insured women using contraceptives.

## Methods

### Data source

This analysis was conducted using healthcare commercial claims from the Optum Health Reporting and Insights database encompassing the time period between January 1999 and March 2014. This database includes administrative claims for over 18.5 million privately insured individuals (17.1 million under age 65) covered by 84 self-insured Fortune 500 companies with locations in all areas of the United States. It contains eligibility information, some demographic characteristics such as gender, region, and salary for employees, along with complete medical and pharmaceutical claims for all of the 84 companies’ beneficiaries (i.e., employees, spouses, dependents, and retirees) nationwide. The data are de-identified and comply with the Health Insurance Portability and Accountability Act (HIPAA) of 1996 to preserve patient anonymity and confidentiality.

### Study design and patient selection

A retrospective longitudinal observational study design was used. Women aged 15 to 44 years old at the time of their SARC or LARC claim were selected. The study period spanning from January 1999 through March 2014 was broken down into six and 12 month intervals for the evaluation of the study endpoints. Some of the analyses conducted required women having at least 12 months of continuous health plan eligibility before a LARC or SARC claim. The Statistical Analysis Section describes the analyses for which this selection criterion was applied.

### Study endpoints

The study endpoints were the use of SARC or LARC in a given semester (semesters are herein defined as half-years, running either from January 1 of a given year up until June 30 of the same year, or from July 1 of a year up until December 31 of the same year) or in a given year. Women were classified as SARC users in a semester if they had at least 60 days of supply of SARC in that semester, unless they initiated a LARC episode in the same semester, in which case they were classified as LARC users. LARC episodes were defined as the period starting with a claim for a LARC device and ending with the earliest of a removal, a pregnancy, an abortion, the use of another contraceptive (SARC with more than 60 days of supply in the semester or permanent sterilization), up to 5 years (Mirena users), up to 3 years (Implanon/Nexplanon and Skyla users), up to 10 years (ParaGard users), a procedure that led to sterilization (i.e., hysterectomy, oophorectomy, salpingectomy), end of age eligibility criterion, end of health plan eligibility, or the end of the data. Women were classified as LARC users in a given semester if that semester overlapped a LARC episode, unless the episode ended with SARC use, in which case they were classified as SARC users. For the assessment of the evolution of the proportion of LARC users over time, LARC and SARC use was evaluated yearly using the same definition.

A sensitivity analysis was also conducted using a 30 days of supply threshold to identify SARC users (instead of 60 days of supply).

### Statistical analysis

#### Baseline characteristics

Descriptive statistics were used to summarize the patient characteristics assessed during the baseline periods for the population having at least 12 months of continuous health plan eligibility before a LARC or SARC claim. Mean, standard deviation, and median were reported for descriptive statistics of continuous variables, and absolute and relative frequency counts were reported for categorical variables.

#### Evolution of the proportion of LARC users over time

The proportion of women using LARC over women using LARC or SARC was reported on a yearly basis during the period 1999–2013. Proportions of LARC over LARC and SARC use were reported each year by age category (i.e., 15–17 years, 18–24 years, 25–34 years, and 35–44 years). Statistical significance between consecutive years was assessed using a Pearson chi-squared test. Of note, this analysis was conducted on a larger sample size because it did not require a population having at least 12 months of continuous health plan eligibility for the assessment of characteristics prior to the use of a SARC or LARC.

#### Predictors of LARC use over time

Generalized estimating equations (GEE) with logit link for binary outcomes and adjusting for repeated measurement every semester in patients who had at least 12 months of continuous eligibility before a SARC or LARC claim were conducted to obtain odds ratios (ORs) and 95 % confidence intervals (CIs). The GEE approach was chosen to account for the longitudinal and correlated nature of repeated semi-annual measurements, particularly relevant in the case of LARCs that lasted from 6 semesters for the shortest duration devices (Implanon, Nexplanon and Skyla) up to 20 for the longest (ParaGard). Patient characteristics evaluated during the previous semester and used as predictors were age group, region, insurance plan type, medical comorbidities, nulliparous status, and gynecologic history. Dichotomous variables identifying the time periods associated with the introduction of newer LARC methods (i.e., 1999Q1-2000Q4, 2001Q1-2006Q2, 2006Q3-2012Q4, and 2013Q1-2014Q1) were also used as predictors in order to identify the impact of the introduction of newer LARC methods (i.e., Mirena in 2001, Implanon/Nexplanon in 2006, and Skyla in 2013) on LARC use. Because the information on the type of industry and on wages is only available for the primary plan holders (i.e., employees) in the Optum Health Reporting and Insights database, a sensitivity analysis was also conducted on the subset of women employees to allow for the use of the industry of employment and wages to be added as covariates in the GEE model.

Finally, a GEE including interaction terms between age groups (i.e., 15–17 years, 18–24 years, 25–34 years, and 35–44 years) and the time periods related to introduction of new LARCs were conducted to identify the evolution in LARC use after introduction of new LARC methods among the different age groups.

## Results

### Population baseline characteristics

**Fig. 1:**
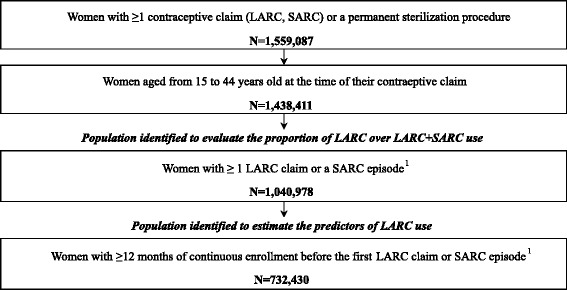
Contraceptive users’ disposition. LARC: Long Acting Reversible Contraceptive, SARC: Short Acting Reversible Contraceptive. *Note*: 1. A SARC episode was defined as SARC claims totaling 60 days of supply or more in a given 6-month period

A total of 1,040,978 women were identified to assess the evolution of proportion of LARC use over time (Fig. 1), while 732,430 women were selected for analyses that used information collected prior to contraceptive claims (12 months of continuous health plan eligibility before a LARC or SARC claim).

Table 1 presents the characteristics of the latter population at the time of a first SARC or LARC claim preceded with more than 12 months of continuous eligibility, in each period corresponding to the introduction of a newer LARC method (i.e., 1999Q1-2000Q4, 2001Q1-2006Q2, 2006Q3-2012Q4, and 2013Q1-2014Q1). Across periods, women opting for LARC were on average older by a few years than those choosing SARC (32.5 vs 30.0 for women in 1999–2000, 32.8 vs 28.8 in 2001–2006, 31.4 vs 27.4 in 2006–2012, and 30.3 vs 27.2 in 2013–2014). Also, a large majority of women using LARC already had children (as per the number of dependents identified under the same insurance plan). However, the proportion of nulliparous LARC users increased since 2001 (14.2 % in 2001–2006, 29.2 % in 2006–2012, and 44.6 % in 2013–2014). Moreover, the plan type increased over time in the proportion of women covered by a point of service (POS) plan (from ~35 % in 1999–2000 to ~63 % in 2013–2014) and decreased in the proportion of women covered by a preferred provider organization (PPO) plan (from ~34 % in 1999–2000 to ~15 % in 2013–2014).

**Table 1:** Baseline Characteristics of Women Using SARC or LARC Method Stratified by Time Period^a^

	Jan 1999–Dec 2000		Jan 2001–Jun 2006		Jul 2006–Dec 2012		Jan 2013–Mar 2014	
	LARC N = 94	SARC N = 17,120	LARC N = 6,379	SARC N = 218,315	LARC N = 49,903	SARC N = 474,109	LARC N = 18,242	SARC N = 214,279
Age at index date, mean ± SD [median]	32.5 ± 6.4 [34]	30.0 ± 7.7 [30]	32.8 ± 5.9 [33]	28.8 ± 8.0 [29]	31.4 ± 7.2 [32]	27.4 ± 8.1 [26]	30.3 ± 7.7 [31]	27.2 ± 7.8 [25]
Age categories, N (%)								
15–17 years	1 (1.1 %)	564 (3.3 %)	23 (0.4 %)	12,799 (5.9 %)	1,106 (2.2 %)	45,554 (9.6 %)	484 (2.7 %)	14,066 (6.6 %)
18–24 years	13 (13.8 %)	4,385 (25.6 %)	549 (8.6 %)	65,033 (29.8 %)	8,723 (17.5 %)	156,526 (33.0 %)	4,809 (26.4 %)	85,938 (40.1 %)
25–34 years	42 (44.7 %)	6,771 (39.6 %)	3,220 (50.5 %)	79,990 (36.6 %)	21,846 (43.8 %)	163,430 (34.5 %)	6,838 (37.5 %)	69,339 (32.4 %)
35–44 years	38 (40.4 %)	5,400 (31.5 %)	2,587 (40.6 %)	60,493 (27.7 %)	18,228 (36.5 %)	108,599 (22.9 %)	6,111 (33.5 %)	44,936 (21.0 %)
Region, N (%)								
South	29 (30.9 %)	8,584 (50.1 %)	2,502 (39.2 %)	86,066 (39.4 %)	16,687 (33.4 %)	149,431 (31.5 %)	5,561 (30.5 %)	58,042 (27.1 %)
Northeast	13 (13.8 %)	2,443 (14.3 %)	899 (14.1 %)	52,203 (23.9 %)	7,474 (15.0 %)	107,570 (22.7 %)	4,520 (24.8 %)	58,460 (27.3 %)
Midwest	29 (30.9 %)	4,342 (25.4 %)	1,008 (15.8 %)	43,311 (19.8 %)	10,099 (20.2 %)	109,389 (23.1 %)	4,256 (23.3 %)	53,605 (25.0 %)
West								
Unknown region	0 (0.0 %)	8 (0.0 %)	93 (1.5 %)	3,299 (1.5 %)	4,267 (8.6 %)	24,237 (5.1 %)	351 (1.9 %)	13,757 (6.4 %)
Employment status, N (%)								
Employee								
Employee	47 (50.0 %)	8,683 (50.7 %)	1,965 (30.8 %)	80,723 (37.0 %)	17,459 (35.0 %)	167,890 (35.4 %)	5,660 (31.0 %)	59,576 (27.8 %)
Retiree	0 (0.0 %)	4 (0.0 %)	63 (1.0 %)	1,990 (0.9 %)	627 (1.3 %)	6,610 (1.4 %)	185 (1.0 %)	2,067 (1.0 %)
Other employee status	5 (5.3 %)	116 (0.7 %)	428 (6.7 %)	6,881 (3.2 %)	3,208 (6.4 %)	12,673 (2.7 %)	1,376 (7.5 %)	10,775 (5.0 %)
Dependant								
Spouse	34 (36.2 %)	4,199 (24.5 %)	3,639 (57.0 %)	62,712 (28.7 %)	20,366 (40.8 %)	105,241 (22.2 %)	5,278 (28.9 %)	34,503 (16.1 %)
Child	8 (8.5 %)	4,042 (23.6 %)	263 (4.1 %)	64,181 (29.4 %)	8,092 (16.2 %)	179,287 (37.8 %)	5,682 (31.1 %)	106,449 (49.7 %)
Other type of dependant	0 (0.0 %)	73 (0.4 %)	14 (0.2 %)	1,615 (0.7 %)	131 (0.3 %)	2,197 (0.5 %)	52 (0.3 %)	803 (0.4 %)
Unknown employee/dependant status	0 (0.0 %)	3 (0.0 %)	7 (0.1 %)	213 (0.1 %)	20 (0.0 %)	211 (0.0 %)	9 (0.0 %)	106 (0.0 %)
Plan type, N (%)								
Point of Service (POS)	26 (27.7 %)	6,040 (35.3 %)	2,561 (40.1 %)	87,078 (39.9 %)	30,094 (60.3 %)	284,798 (60.1 %)	11,289 (61.9 %)	137,026 (63.9 %)
Preferred Provider Organization (PPO)	32 (34.0 %)	5,837 (34.1 %)	1,922 (30.1 %)	69,951 (32.0 %)	7,059 (14.1 %)	76,131 (16.1 %)	2,959 (16.2 %)	32,516 (15.2 %)
Health Maintenance Organization (HMO)	8 (8.5 %)	1,099 (6.4 %)	912 (14.3 %)	30,219 (13.8 %)	5,871 (11.8 %)	55,814 (11.8 %)	1,479 (8.1 %)	19,302 (9.0 %)
Indemnity	9 (9.6 %)	1,298 (7.6 %)	231 (3.6 %)	12,744 (5.8 %)	3,212 (6.4 %)	28,194 (5.9 %)	1,334 (7.3 %)	17,682 (8.3 %)
Unknown plan type	19 (20.2 %)	2,846 (16.6 %)	753 (11.8 %)	18,323 (8.4 %)	3,667 (7.3 %)	29,172 (6.2 %)	1,181 (6.5 %)	7,753 (3.6 %)
Available information on wage and type of industry, N (%)	33 (35.1 %)	6,534 (38.2 %)	2,108 (33.0 %)	78,405 (35.9 %)	18,990 (38.1 %)	170,724 (36.0 %)	6,731 (36.9 %)	65,991 (30.8 %)
Wage at index date, 2014 US$, mean ± SD [median]	59,772 ± 22,281 [57,801]	64,295 ± 42,416 [56,216]	68,794 ± 45,956 [55,170]	60,502 ± 35,707 [51,780]	51,134 ± 38,273 [40,809]	51,730 ± 35,979 [42,714]	47,033 ± 36,759 [34,083]	48,186 ± 34,453 [36,162]
Type of industry, N (%)								
Technology	5 (15.2 %)	2,290 (35.0 %)	218 (10.3 %)	25,574 (32.6 %)	2,774 (14.6 %)	27,472 (16.1 %)	612 (9.1 %)	5,872 (8.9 %)
Transportation	3 (9.1 %)	544 (8.3 %)	270 (12.8 %)	7,753 (9.9 %)	2,975 (15.7 %)	16,368 (9.6 %)	812 (12.1 %)	6,161 (9.3 %)
Financial	9 (27.3 %)	1,835 (28.1 %)	188 (8.9 %)	17,789 (22.7 %)	2,009 (10.6 %)	25,725 (15.1 %)	1,558 (23.1 %)	14,605 (22.1 %)
Manufacture	10 (30.3 %)	1,480 (22.7 %)	141 (6.7 %)	6,238 (8.0 %)	1,169 (6.2 %)	10,319 (6.0 %)	389 (5.8 %)	4,179 (6.3 %)
Consumer	6 (18.2 %)	385 (5.9 %)	62 (2.9 %)	6,181 (7.9 %)	2,689 (14.2 %)	28,510 (16.7 %)	572 (8.5 %)	9,661 (14.6 %)
Government	0 (0.0 %)	0 (0.0 %)	193 (9.2 %)	3,216 (4.1 %)	2,941 (15.5 %)	18,085 (10.6 %)	952 (14.1 %)	8,362 (12.7 %)
Healthcare	0 (0.0 %)	0 (0.0 %)	143 (6.8 %)	5,729 (7.3 %)	829 (4.4 %)	9,502 (5.6 %)	253 (3.8 %)	2,315 (3.5 %)
Other	0 (0.0 %)	0 (0.0 %)	893 (42.4 %)	5,925 (7.6 %)	3,604 (19.0 %)	34,743 (20.4 %)	1,583 (23.5 %)	14,836 (22.5 %)
Comorbidities, N (%)								
Asthma	1 (1.1 %)	498 (2.9 %)	271 (4.2 %)	7,726 (3.5 %)	2,399 (4.8 %)	20,858 (4.4 %)	924 (5.1 %)	10,684 (5.0 %)
Hypothyroidism	1 (1.1 %)	362 (2.1 %)	339 (5.3 %)	6,491 (3.0 %)	2,633 (5.3 %)	16,640 (3.5 %)	917 (5.0 %)	8,616 (4.0 %)
Hypertension	4 (4.3 %)	309 (1.8 %)	341 (5.3 %)	4,663 (2.1 %)	2,568 (5.1 %)	12,099 (2.6 %)	850 (4.7 %)	5,515 (2.6 %)
Diabetes	3 (3.2 %)	136 (0.8 %)	113 (1.8 %)	2,283 (1.0 %)	936 (1.9 %)	5,775 (1.2 %)	323 (1.8 %)	2,609 (1.2 %)
Obesity	0 (0.0 %)	132 (0.8 %)	120 (1.9 %)	2,511 (1.2 %)	1,899 (3.8 %)	8,900 (1.9 %)	921 (5.0 %)	5,445 (2.5 %)
Inflammatory bowel disease	0 (0.0 %)	59 (0.3 %)	22 (0.3 %)	882 (0.4 %)	257 (0.5 %)	2,129 (0.4 %)	124 (0.7 %)	1,217 (0.6 %)
Epilepsy	1 (1.1 %)	57 (0.3 %)	66 (1.0 %)	857 (0.4 %)	533 (1.1 %)	2,538 (0.5 %)	220 (1.2 %)	1,446 (0.7 %)
Rheumatoid arthritis	0 (0.0 %)	47 (0.3 %)	30 (0.5 %)	659 (0.3 %)	218 (0.4 %)	1,741 (0.4 %)	107 (0.6 %)	954 (0.4 %)
Venous thromboembolism	1 (1.1 %)	20 (0.1 %)	58 (0.9 %)	293 (0.1 %)	411 (0.8 %)	681 (0.1 %)	142 (0.8 %)	300 (0.1 %)
Systemic lupus erythematosus	0 (0.0 %)	19 (0.1 %)	18 (0.3 %)	297 (0.1 %)	174 (0.3 %)	704 (0.1 %)	43 (0.2 %)	326 (0.2 %)
Patients switching contraception method^b^, N (%)	3 (3.2 %)	13 (0.1 %)	532 (8.3 %)	2,357 (1.1 %)	5,404 (10.8 %)	17,501 (3.7 %)	1,284 (7.0 %)	3,948 (1.8)
Gynaecology history^c^, N (%)								
History of SARC use	23 (24.5 %)	11,542 (67.4 %)	2,225 (34.9 %)	138,290 (63.3 %)	15,857 (31.8 %)	309,903 (65.4 %)	5,753 (31.5 %)	172,756 (80.6 %)
History of LARC use	1 (1.1 %)	15 (0.1 %)	187 (2.9 %)	541 (0.2 %)	2,355 (4.7 %)	4,006 (0.8 %)	1,472 (8.1 %)	2,760 (1.3 %)
History of obstetrician/gynaecologist visits	80 (85.1 %)	9,339 (54.6 %)	5,875 (92.1 %)	138,829 (63.6 %)	44,833 (89.8 %)	303,585 (64.0 %)	15,684 (86.0 %)	140,701 (65.7 %)
Pregnancy history	44 (46.8 %)	1,671 (9.8 %)	3,182 (49.9 %)	25,803 (11.8 %)	21,556 (43.2 %)	49,069 (10.3 %)	5,895 (32.3 %)	16,885 (7.9 %)
Abortion history	6 (6.4 %)	145 (0.8 %)	267 (4.2 %)	2,194 (1.0 %)	1,577 (3.2 %)	4,906 (1.0 %)	505 (2.8 %)	1,587 (0.7 %)
Nulliparous status, N (%)								
Nulliparous	21 (22.3 %)	10,116 (59.1 %)	903 (14.2 %)	131,780 (60.4 %)	14,571 (29.2 %)	319,441 (67.4 %)	8,136 (44.6 %)	158,741 (74.1 %)
Non-nulliparous	73 (77.7 %)	6,928 (40.5 %)	5,455 (85.5 %)	84,707 (38.8 %)	35,181 (70.5 %)	152,260 (32.1 %)	10,045 (55.1 %)	54,629 (25.5 %)
Unknown nulliparous status	0 (0.0 %)	76 (0.4 %)	21 (0.3 %)	1,828 (0.8 %)	151 (0.3 %)	2,408 (0.5 %)	61 (0.3 %)	909 (0.4 %)

^a^Baseline characteristics of patients with either a LARC claim or claims totaling 60 days of supply of SARC. The 12-month period before the first SARC or LARC claims for each patient in each time period was used to calculate the characteristics

^b^Patients switching from a SARC to a LARC or from a LARC to a SARC during the time period where the characteristics were evaluated

^c^Evaluated during the 12-month baseline period

### Evolution of the proportion of LARC users over time

**Fig. 2:**
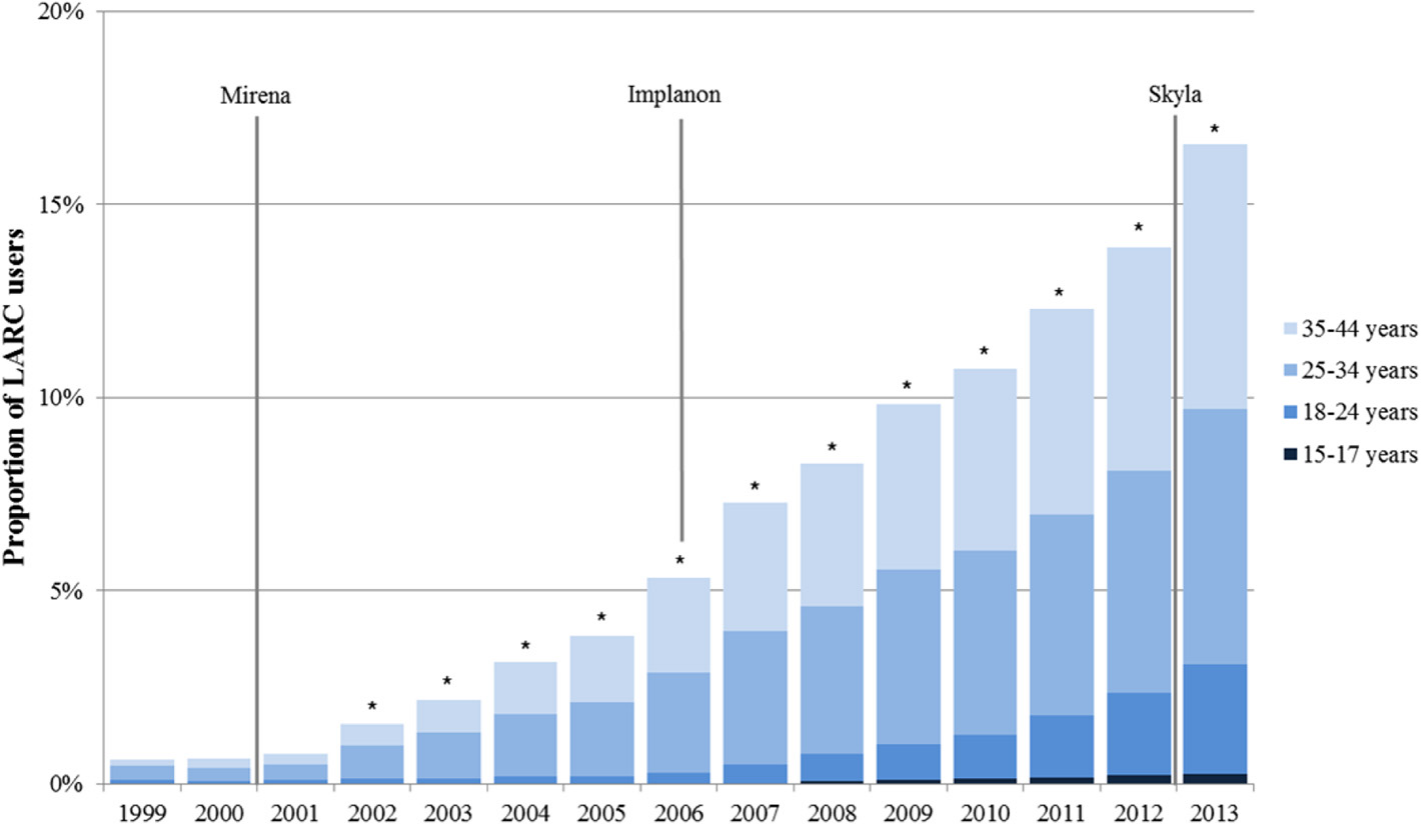
Progression of LARC use over time among LARC and SARC users. * Denotes statistical significance (*P* < 0.05) compared to the previous year using a Pearson chi-squared test. LARC: Long Acting Reversible Contraceptive, SARC: Short Acting Reversible Contraceptive

Figure 2 shows the evolution of the proportion of women using LARC among women using LARC or SARC over the time period encompassing 1999–2013. The proportion increased consistently from 0.61 % in 1999 up to 16.58 % in 2013, and the difference in proportions compared to the previous year was statistically significant for each year after 2001. The figure also shows a majority of women were 25–44 years old, and an acceleration in adoption of LARC methods among younger women, particularly in the group 18–24.

In the sensitivity analysis using a 30 days of supply threshold to identify SARC users, the results remained consistent (data not shown).

### Predictors of LARC use over time

Table 2 displays the estimated odds of using LARC among the population who used LARC or SARC from 1999 through 2014 and among the subset of women employees. Time periods related to the introduction of newer LARC methods were significant predictors of LARC use, with women before 2001 being 0.28-fold (95 % CI: 0.24-0.33) less likely to use LARC than women in the period 2001–2006, women in the period 2006–2012 being 3.66-fold (95 % CI: 3.57-3.74) more likely to use LARC than women in the period 2001–2006, and women in the period 2013–2014 being 6.61-fold (95 % CI: 6.43-6.80) more likely to use LARC than women in 2001–2006.

**Table 2:** Predictors of LARC use among women using SARC or LARC

	Odds Ratio^a^ (95 % CI)	
	All Women (N = 732,430)	All Employees^b^ (N = 249,021)
Time Period		
Jan 1999–Dec 2000	0.28 (0.24 - 0.33)*	0.24 (0.18 - 0.33)*
Jan 2001–Jun 2006	1.00	1.00
Jul 2006–Dec 2012	3.66 (3.57 - 3.74)*	3.63 (3.47 - 3.80)*
Jan 2013–Mar 2014	6.61 (6.43 - 6.80)*	6.73 (6.37 - 7.10)*
Age group		
15–17 year old	0.55 (0.53 - 0.58)*	-
18–24 year old	1.00	1.00
25–34 year old	1.03 (1.00 - 1.05)*	1.29 (1.22 - 1.37)*
35–44 year old	1.21 (1.18 - 1.24)*	1.63 (1.54 - 1.73)*
Region		
South	1.00	1.00
Northeast	0.80 (0.79 - 0.82)*	0.88 (0.84 - 0.92)*
Midwest	0.90 (0.88 - 0.92)*	0.97 (0.93 - 1.00)
West	1.27 (1.24 - 1.30)*	1.29 (1.24 - 1.34)*
Unknown region	1.39 (1.35 - 1.44)*	1.20 (1.12 - 1.29)*
Plan type		
Point of Service (POS)	1.00	1.00
Preferred Provider Organization (PPO)	0.98 (0.95 - 1.01)	0.89 (0.85 - 0.94)*
Health Maintenance Organization (HMO)	1.05 (1.03 - 1.08)*	0.93 (0.90 - 0.97)*
Indemnity	1.14 (1.10 - 1.18)*	1.04 (0.98 - 1.12)
Unknown plan type	1.21 (1.17 - 1.25)*	1.31 (1.23 - 1.38)*
Type of industry		
Technology	-	1.00
Transportation	-	1.44 (1.36 - 1.52)*
Financial	-	0.93 (0.88 - 0.98)*
Manufacture	-	1.07 (1.00 - 1.14)*
Consumer	-	1.07 (1.01 - 1.13)*
Government	-	1.57 (1.47 - 1.67)*
Healthcare	-	0.81 (0.75 - 0.87)*
Other	-	1.07 (1.02 - 1.12)*
Wage (per 100,000 2014 US$)	-	0.94 (0.90 - 0.98)*
Comorbidities		
Venous thromboembolism	13.50 (6.82 - 26.72)*	22.12 (7.77 - 62.93)*
Systemic lupus erythematosus	1.90 (1.58 - 2.28)*	1.92 (1.42 - 2.59)*
Epilepsy	1.92 (1.02 - 3.60)*	1.39 (0.45 - 4.31)
Hypertension	1.55 (1.48 - 1.63)*	1.63 (1.51 - 1.75)*
Obesity	1.54 (1.46 - 1.64)*	1.64 (1.50 - 1.79)*
Asthma	1.25 (1.19 - 1.31)*	1.30 (1.19 - 1.41)*
Diabetes	1.17 (1.09 - 1.26)*	1.21 (1.08 - 1.36)*
Rheumatoid arthritis	1.19 (1.04 - 1.35)*	1.22 (0.97 - 1.54)
Inflammatory bowel disease	1.09 (0.95 - 1.24)	1.17 (0.94 - 1.45)
Hypothyroidism	1.05 (1.00 - 1.10)*	1.12 (1.04 - 1.21)*
Gynecology history		
History of SARC use	0.08 (0.08 - 0.08)*	0.07 (0.07 - 0.07)*
History of LARC use	254.14 (248.85 - 259.55)*	310.20 (297.55 - 323.39)*
History of obstetrician/gynecologist visits	1.75 (1.72 - 1.78)*	1.79 (1.74 - 1.85)*
Pregnancy history	2.80 (2.74 - 2.85)*	2.32 (2.24 - 2.41)*
Abortion history	0.98 (0.94 - 1.01)	0.93 (0.87 - 1.00)*
Nulliparous status		
Nulliparous	1.00	1.00
Non nulliparous	2.29 (2.24 - 2.34)*	2.56 (2.48 - 2.64)*
Unknown nulliparous status	1.30 (1.15 - 1.47)*	-

^a^Odds ratios and confidence intervals were obtained using a generalized estimating equation model controlling for all variables listed and adjusting for repeated measurements among patients

^b^Women aged 18 or more with employee status and for whom wage was known were considered for this analysis

*Denotes statistical significance compared to the reference (*P*<0.05)

Age group was another significant predictor of LARC use. Compared to women aged 18–24 years, women aged 15–17 years were 0.55-fold (95 % CI: 0.53-0.58) less likely to use LARC, women aged 25–34 years were 1.03-fold (95 % CI: 1.00-1.05) more likely to use LARC, and women aged 35–44 were 1.21-fold (95 % CI: 1.18-1.24) more likely to use LARC.

Regarding clinical factors, women with venous thromboembolism (VTE) were 13.50-fold (95 % CI: 6.82-26.72) more likely to use LARC compared to women without VTE. All other comorbidities (i.e., systemic lupus erythematous, epilepsy, hypertension, obesity, asthma, diabetes, rheumatoid arthritis, and hypothyroidism), except inflammatory bowel disease, were associated with a higher likelihood of LARC use. Parous women were 2.29-fold (95 % CI: 2.24-2.34) more likely than nulliparous women to use LARC.

Results of the sensitivity analysis on the subset population of women employees were very similar to the results described above (Table 2). In the sensitivity analysis using a 30 days of supply threshold to identify SARC users, the results were consistent (data not shown).

#### Influence of age groups and time periods over LARC use

**Fig. 3:**
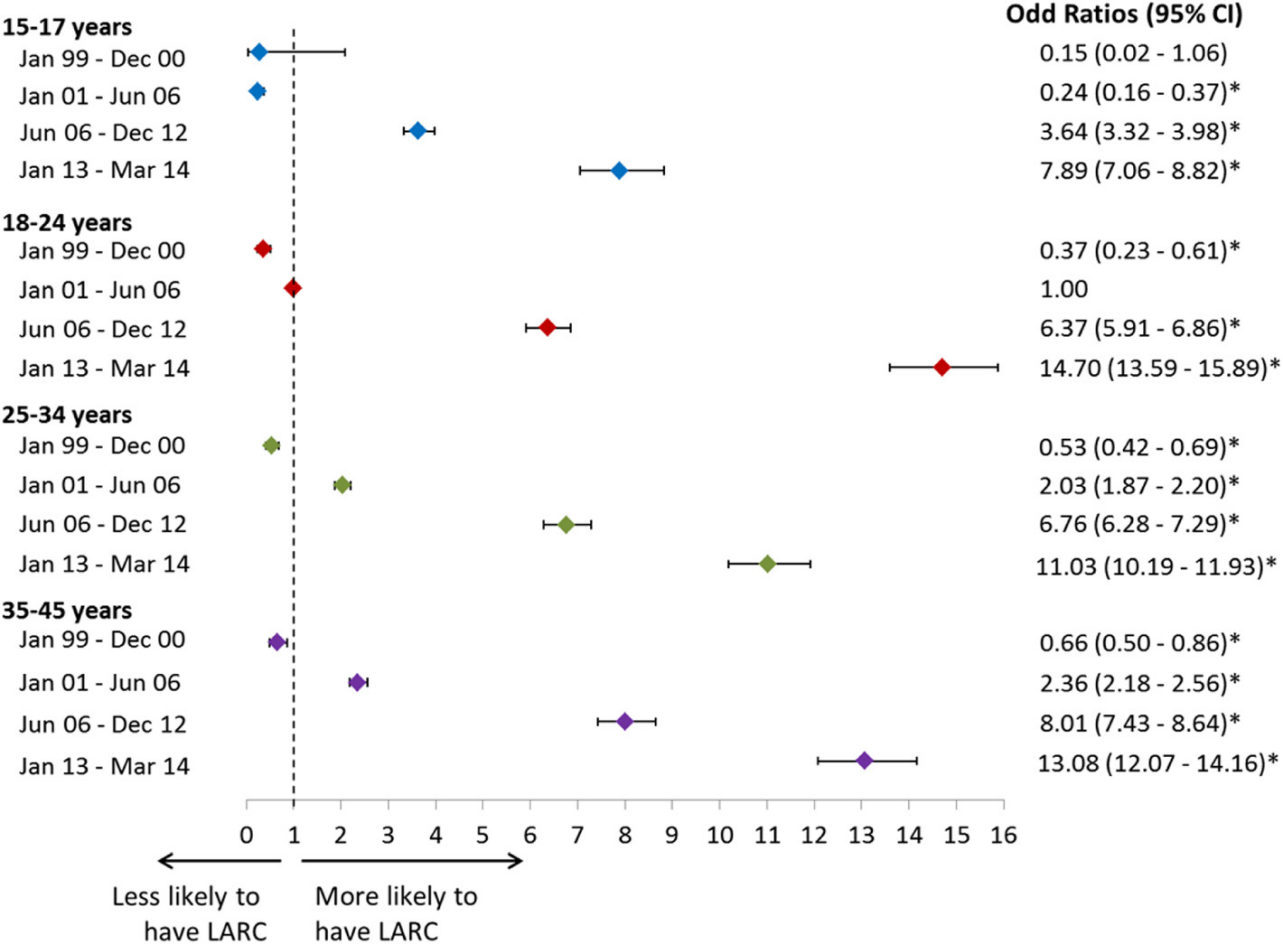
Likelihood of Using LARC (over SARC) Across Time Periods and Age Groups [1]. * Denotes statistical significance (*P* < 0.05) compared to the reference group of women 18–24 years old using LARC in the period January 2001 – June 2006. LARC: Long Acting Reversible Contraceptive, SARC: Short Acting Reversible Contraceptive. *Note*: The following covariates were also included in the model: region, plan type, comorbidities (i.e., venous thromboembolism, systemic lupus erythematosus, epilepsy, hypertension, obesity, asthma, diabetes, rheumatoid arthritis inflammatory bowel disease, and hypothyroidism), history of SARC use, history of LARC use, history of obstetrician/gynecologist visits, pregnancy history, abortion history, and nulliparous status

Figure 3 shows the odds ratios (95 % CI) of using LARC for age group and time period interaction terms. Across all age groups, women in the most recent period (i.e., 2013–2014) had a higher likelihood of using LARC as opposed to women of the same age in more recent periods (i.e., 1999–2000, 2001–2006, and 2006–2012). The increase in LARC use was especially pronounced in young women. Compared to women aged 18–24 in 2001-2006, women aged 18–24 in 2006-2012 and in 2013-2014 were respectively 6.4-fold (95%CI: 5.91-6.86) and 14.7-fold (95%CI: 13.59-15.89) more likely to use LARC over SARC methods.

## Discussion

This large retrospective study based on commercial healthcare claims data found that the use of LARC methods among US women seeking contraception increased between January 1999 and March 2014. Moreover, the observation held for all age groups among women of childbearing age (15–44 years old) and also suggested that the introduction of new LARC products and consequently the potential fulfillment of women’s unmet contraceptive needs, could be an important driver of LARC use. The current study has the advantage of relying on a large sample of women insured through their employer. These employers, part of the Fortune 500 ranking, are likely to offer advantageous health plan coverage and to cover most contraceptives including LARCs. This study thus suggests that maintaining an open-access to all products and providing more options within the LARC category could enable more women to choose a LARC over a SARC method.

The statistically significant increase in LARC use since 2002 reported in this study concurs with the results of the recent US National Survey of Family Growth (NSFG) that reported that LARC use among all women 15–44 years old had increased from 1.5 % in 2002 to 7.2 % in the period 2011–2013 [9]. Another analysis of the 2002 and 2006–2010 NSFG data previously demonstrated that the proportion of LARC users among all contraceptive users had grown from 2.4 % in 2002 up to 8.5 % in 2009 [11]. One of the reasons for this evolution may be a shift in reproductive health expert opinions and recommendations regarding LARCs. IUDs in particular were traditionally seen as appropriate for parous women only, in part owing to label recommendations to that effect which were initially present for ParaGard and Mirena, the only two IUDs on the market in the US for quite some time following the Dalkon shield debacle. The recommendation remains for Mirena but was removed from ParaGard labeling in 2005 [15]. Skyla and Liletta, approved by the US food and drug administration (FDA) in 2013 and 2015, respectively, included nulliparous subjects in the registration clinical trials and consequently do not contain such a recommendation; rather, the labeling specifies that they are indicated for prevention of pregnancy without reference to any particular population [16, 17]. The former is included in the time frame of this study while the latter is not due to its very recent approval.

Contrary to the past perceptions and opinions, LARC methods are safe for nulliparous young women, do not cause tubal infertility, and studies report a rapid return to fertility after removal [18–20]. Consequently, experts have been increasingly advocating for use of LARC methods prior to childbearing [21–23]. Hence the American Congress of Obstetricians and Gynecologists recommended IUDs as first-line contraception as early as 2005, for users including teenagers and younger women, and the American Academy of Pediatrics has recently promulgated the same recommendation [21, 22]. More recently, the Centers for Disease Control and prevention reported that IUDs were safe and effective in nulliparous women [23].

However, there are still several obstacles hindering LARC use. One of them is perceived high costs [13, 14], although it has been proven that LARC methods are highly cost-effective when used for as little as the shortest available duration of three years [24, 25]. The Affordable Care Act (ACA) of 2010 that imposes on most healthcare plans to cover the full range of contraceptive methods as of August 2012, including LARC, with no patient cost-sharing, could weaken this barrier [11]. Unfortunately, for many plans, the requirement only took effect in 2013; furthermore, many exemptions relative to contraception coverage were granted; and finally, a 2014 ruling of the U.S. Supreme Court allowed closely held, for-profit firms to opt out of the contraceptive coverage mandate in the ACA [26]. In relation to the ACA, the Guttmacher Institute reported that several plans currently cover prescription contraceptive drugs but not devices [26]. The impact of the ACA on promoting LARC adoption remains therefore unclear, and it is specifically difficult to control for such an event given that its implementation has been made over such a long time span. Another barrier to increasing LARC use is lingering myths and misconceptions about LARC and contraception, at both the user and provider level [27, 28]. Russo et al. [29] recently reviewed and attempted to debunk such misconceptions (e.g., IUDs cause pelvic inflammatory disease) in their 2013 stud. Meanwhile, it was also found in a 2012 study that patients overestimated SARC method efficacy [30]. Therefore, there is still a need to better educate women and providers on contraception methods and LARC in particular.

Broadening the choice of LARC methods could also help increase LARC use, and potentially contribute to changing the perceptions of women and healthcare providers about them. Indeed, bringing to market new devices could help increase the visibility of LARC methods by generating promotional activity from the manufacturer which in turn can raise awareness among both consumers and providers, and by increasing the research interest for LARC, further contributing to an increased knowledge around that class of contraceptives. Diversifying LARC options could also help address the variety of needs of women seeking mid-to-long term reversible contraception [31]. This is supported by the results of the present study, which showed that periods following introduction of newer LARC methods were one of the main drivers of LARC use.

The present study also showed an acceleration of LARC adoption among younger women since availability of Skyla in 2013. Notably, women from the 18–24 age group were 14.7-fold more likely to use a LARC method compared to women of the same age group in 2001-2006. In addtition, that likelihood was also higher than that of women from the 25–34 and 35–44 age groups compared to the same 18–24 age group in 2001–2006 (respectively 11.03 and 13.08). This could be related to the change in reproductive health specialists’ opinions and recommendations regarding the suitability of LARCs for younger women already discussed previously [22, 32, 33], but also, as pointed above, to the availability of new LARC products that better address the needs of younger and nulliparous women.

Results of the present study also showed that among common comorbidities associated with childbearing age, VTE history was a strong predictor of LARC use (estimated likelihood of LARC use after a VTE episode was 13.5). As oral contraceptives, are associated with an increased risk of VTE [34], and as some LARC methods, such as Mirena, decrease or can stop menstrual bleeding, they can be a safer option for VTE patients who have to be treated with blood thinning medication, and for whom pregnancy is a risk factor for VTE (VTE being the leading cause of maternal death in the US) [35]. More generally, LARC methods increase and enhance the spectrum of contraceptive options in women with medical conditions (e.g., systemic lupus erythematosus), either to more efficiently prevent pregnancy or because the condition treatment would interfere with traditional contraception (e.g., epileptic treatment interfering with hormonal pills) [36].

### Limitations

This study is subject to certain limitations. First, even though an algorithm was developed to capture the end of a LARC episode, all removal of LARC methods may not have been captured. Second, claims databases may contain inaccuracies or omissions in procedures, diagnoses, or costs, and no information was provided as to whether or not medication was taken as prescribed. Third, contraceptives purchased over-the-counter were not available in the database, which may have resulted in an underestimation of SARC use. Fourth, even though the Optum Health Reporting and Insights database can be generalized to the large subgroup of the employed US population, our study population consisted of privately-insured individuals, and, therefore, our results might not be generalizable to the entire population of contraceptive users of which an important proportion is uninsured or publicly-insured. Fifth, it is difficult to ignore the influence of the growing familiarity of the public with LARC on LARC adoption, or assess whether this growth would have happened at all without the introduction of new LARC methods that fulfills women’s unmet contraceptive needs. Nonetheless, we think both factors played an important role in the increasing popularity of the use of LARC. Finally, public discussions of benefits and risks of the different contraceptives (or other events such as changes in health insurance coverage and re-imbursement rules or in perception of the suitability of IUDs for nulliparous or younger women) may influence the prescription of LARC and SARC methods; however, time periods associated with these discussions and events were not considered as specific covariates in the statistical models used in this study. These factors may have been captured nonetheless in the time trend that was included as a covariate in the study.

## Conclusions

This study found that privately insured women of childbearing age seeking contraception increasingly turned towards LARC methods over SARC methods from 1999 to 2014. It also found that one of the main associations of this trend was the availability of newer, more diversified, LARC methods, and that the rising trend in LARC use was particularly pronounced among younger women. With the still preoccupyingly high rate of unintended pregnancies in the US, the high costs associated with unintended pregnancies, and the difficulties often faced by women seeking abortion, this study supports the possibility that a sustained effort in broadening the range of available LARC methods may facilitate their adoption. Future research is needed to assess LARC use in uninsured or publicly-insured populations.

## Abbreviations

ACA, affordable care act; CI, confidence interval; FDAl, US Food and Drug Administration; GEE, generalized estimating equation; IUD, intrauterine device; LARC, long acting reversible contraceptive; NSFG, national survey of family growth; OR, odds ratio; SARC, short acting reversible contraceptive; US, United States; VTE, venous thromboembolism
